# A Case of Pregnancy and Parturition during the Existence of Cancer of the Uterus

**Published:** 1847-05

**Authors:** Joseph A. Eve

**Affiliations:** Professor of Obstetrics, &c., &c., in the Medical College of Georgia


*A Case of Pregnancy and Parturition during the existence of Cancer of the Uterus.* By Joseph A. Eve, M. D., Professor of Obstetrics, &c., &c., in the Medical College of Georgia.—Carcinoma of the uterus was formerly supposed to be incompatible with pregnancy; but the possibility of this complication with this disease in all its stages is acknowledged, and its influence in hastening its progress admitted, by all or nearly all authors who have recently written on the diseases of females. A record of cases, or an allusion to this unfortunate complication, will be found in the works of Clarke, Davis, Ashwell, Churchill, Ramsbotham, Waller, Ferguson, Duparcque, Colombat, Boivin & Duges, Velpeau, Siebold, and many others. But it is, in an excellent practical treatise on Organic Diseases of the Womb, by Mr. Lever, of London, that we find the most satisfactory account of pregnancy in connection with cancer, and the most extensive reference to cases.

Pregnancy and cancer have each a prejudicial influence over the other—the former hastening the progress and fatal termination of the latter; and the latter in a considerable number, I believe in a large majority of cases, causing either an abortion or the death of the fœtus when delivery occurs at the full term. The fœtus sometimes perishes in utero, its farther development being prevented, and abortion the necessary consequence; in other instances the death of the fœtus is the result of impediment to delivery, from the schirrous enlargement of the mouth or neck of the uterus.

Of one hundred and twenty cases of malignant disease of the uterus, referred to by Mr. Lever, abortion occurred in forty per cent.; in twenty-seven of delivery, fifteen children were born dead, ten living, and in two the result was not known, or we may say fifteen out of twenty-five were lost.

The object of the present communication is to give a brief history of a remarkable case of pregnancy and parturition in connexion with cancer of the uterus.

July 28th, 1845. I was called in haste, eleven or twelve miles in the country, to visit Mrs. ———, who I was informed had been some time in violent labour. There was considerable time lost in consequence of my absence from town. On my first examination I found the head very low in the pelvis, the mouth of the womb extinguished, except a small portion, which had a tumid, hard, rough, unnatural feel. This labor was far more difficult, painful, and protracted than her two preceding labours, in which I had attended her. The child was expelled about a half hour after my arrival. I remarked a smell very similar to that of cancer of the womb, but did not at the time suppose it possible that it could be identical with it, for she was apparently in most excellent health, remarkably robust and stout, weighing not less than two hundred and fifty pounds, and being about twenty-eight years old, and furthermore, as the child to which she gave birth was large and healthy.

Two or three months previous to her confinement, I was consulted by her family physician in reference to a sanguine discharge to which she had been subject for some time, and which I feared might depend on placenta prævia, but which I have no doubt now was consequent on carcinoma. I have since learned, upon enquiry, that as early as January, she complained of severe pains in the region of the uterus, and that in the very commencement of gestation she experienced unusual sensations that caused her for a long time to doubt whether she was pregnant.

After her confinement Mrs. ——— had an offensive discharge from the vagina. On expressing the opinion, when consulted in reference to it, that she was laboring under organic disease of the uterus, I was requested to visit her, October 5th, with a professional friend, and make an examination with the speculum.

The touch discovered an extensive schirrous enlargement of the neck of the uterus. We could not determine satisfactorily the extent of the ulceration by the speculum, because, before we could make a proper inspection, we were compelled to remove the speculum, for she became so excessively agitated that we feared an hysteric convulsion would have been induced.

As she was young and remarkably robust, we considered this was a case in which every possible effort should be made, although even under such favorable circumstances we had scarcely the slightest shadow of hope—favorable, I mean, in reference to her age, constitution and general health, but quite the contrary when viewed with respect to her recent gestation.

We put her on the internal use of proto-iodide of mercury, and chloride of soda as a vaginal injection, with an occasional resort to the sulphate of morphine, whenever pain might call it into requisition ; she was however at this time, and for a considerable time after, comparatively free from suffering. We proposed to apply some cautery, at another visit, when she might be sufficiently composed to bear its application, either the nitrate of silver or nitrate of mercury.

I was requested to visit her again, the 21st of the same month, sixteen days after my first visit. She had not yet lost her embonpoint, but the cancerous ulceration had made most frightful and destructive progress, having involved not only the posterior lip, but the posterior part of the cervix or body. It was now too late to think of anything beyond palliative measures. We advised a lotion of the nitrate of silver, with the view of correcting the fetor and improving the condition of the ulcers, perhaps in some degree checking their course. After this she became subject to most alarming and exhausting hæmorrhages at each menstrual period. She now began to lose flesh, and strength rapidly, and to suffer severe lancinating pains.

I visited her again the 6th of November. The disorganization was still more rapid, far exceeding anything I had ever before witnessed. We endeavored to support her strength by tonics, to alleviate her sufferings by opiates, to restrain the hæmorrhages by styptics and astringent lotions, and to correct the horrible fetor by the chloride of soda.

After the destruction of the posterior lip, posterior portion of the neck and body of the uterus, the ulceration extended through the vagina and rectum, allowing the fæces to pass from the latter through the former, and must have involved even the sacral plexus of nerves from the excruciating paroxysmal pains she suffered in that region.

I never saw her after the 23d December, but was informed by my friend that she continued to linger in the most painful and deplorable condition until the 25th of June, when death kindly released her from sufferings indescribably severe, almost beyond endurance.

It is impossible, from any thing we could learn of the history of this case, to determine how long the schirrus may have preceded the commencement of gestation : it is probable not very long, from the excellence of her general health and the fact that she did not complain of pain, or any unusual sensation in the pelvis, until about the time she became pregnant.

This case is remarkable, from having occured in so young, healthy and robust a subject, from the fact, that the process of gestation was conducted most perfectly, notwithstanding the presence of schirrous certainly, and I think we may safely say cancerous ulceration, from the discharge and the characteristic fetor, parturition only being rendered somewhat more tedious and difficult. But if it is remarkable for the absence of any obvious effect of the cancer on the gestation, it is still more so for the very marked influence of the latter over the former. After delivery, the progress of the disease was extremely rapid, although in the early age, health and vigor of the patient, it might have been expected to have run a slower and longer course.

Mr. Lever considers twenty months to be the usual or average duration of uterine cancer. Dr. Ashwell concurs with him, if he refers, as he doubtless does, to the stage of ulceration. I would suppose, from my own comparatively limited observation, that the ulcerative stage generally lasts at least twenty months. In this case, there intervened only eleven months between her confinement and her death, although she possessed uncommon vigor of constitution and appeared to resist death much longer than any person could have supposed, considering the ravages of the disease and the intensity of her sufferings. I cannot speak positively with respect to the commencement of ulceration : I would infer, from the hæmorrhages during gestation, and the fœtid discharge during labour, that it existed previous to her confinement ; but it certainly had not progressed far, even at my first visit, more than two months afterwards ; it was so superficial that it was not evident to the touch, and, as I have remarked, in consequence of her extreme agitation and excitement, the examination by the speculum was not satisfactory. It is singular that ulceration had made comparatively so little progress, between the time of her confinement and my first visit, and so much between my first and second visit. It is probable if I could have made a satisfactory examination at my first visit, a larger ulcerated surface would have been discovered ; but after making all due allowance, I am confident, it was very limited compared with the progress made at my second visit.

If I had had an opportunity of examining this patient, during gestation, at the commencement of labour, and a month or six weeks after delivery, these details would have been more satisfactory ; but I have related them as particularly as I could under the circumstances. *Southern Med. and Surg. Journ.*

